# Total skin self-examination at home for people treated for cutaneous melanoma: development and pilot of a digital intervention

**DOI:** 10.1136/bmjopen-2015-007993

**Published:** 2015-08-06

**Authors:** Peter Murchie, Julia L Allan, William Brant, Matthew Dennis, Susan Hall, Judith Masthoff, Fiona M Walter, Marie Johnston

**Affiliations:** 1 Academic Primary Care, Division of Applied Health Sciences, University of Aberdeen, Aberdeen, UK; 2 Aberdeen Health Psychology Group, Division of Applied Health Sciences, University of Aberdeen, Aberdeen, UK; 3 Dr Gray's Hospital, Elgin, Moray, UK; 4 dot.rural Digital Economy Hub, University of Aberdeen, King's College, Aberdeen, UK; 5 Department of Computing Science, University of Aberdeen, King's College, Aberdeen, UK; 6 Department of Public Health and Primary Care, Strangeways Research Laboratory, University of Cambridge, Cambridge, UK

## Abstract

**Objectives:**

To develop a digital intervention to prompt, support, and respond to the outcomes of total skin self-examinations (TSSEs) at home by people treated for cutaneous melanoma.

**Design:**

A complex intervention development study.

**Setting:**

Northeast Scotland.

**Participants:**

Semistructured scoping interviews; people previously treated for cutaneous melanoma (n=21). Pilot testing: people treated for melanoma stages 0–2C (n=20); general practitioners (n=6); and a nurse specialist in dermatology (n=1).

**Intervention:**

A tablet-based digital intervention designed to prompt and support TSSEs comprising instructional videos and electronic reporting (including photographs) to a clinical nurse specialist in dermatology, with subsequent clinical triage.

**Primary and secondary outcome measures:**

Qualitative assessment of intervention feasibility and acceptability, and quantitative assessment of intentions and confidence to perform TSSEs in pilot participants.

**Results:**

The majority of pilot participants were strongly positive and adhered well to the intervention (n=15), with 7 of these reporting symptoms of concern at some point during the 6-month pilot. 4 patients complied intermittently, 3 reporting skin problems at least once during the pilot, and 1 withdrew. 2 patients underwent skin surgery as a result of participating in the pilot, with 1 diagnosed as having a recurrent melanoma and the other, a benign lesion. A number of practical issues to improve the usability of the intervention were identified. The proportion of participants reporting intention to check their skin at least monthly increased during the intervention as did confidence to conduct a skin check.

**Conclusions:**

People previously treated for cutaneous melanoma are prepared to use digital technology to support them in conducting TSSE. An intervention has been developed which is practical, effective and safe, and after addressing minor practical issues, could now be evaluated for clinical outcomes in a randomised clinical trial.

**Strengths and limitations of this study**

- The study involved all key stakeholders in melanoma follow-up programmes.
- The study followed a well-evidenced and iterative approach to developing theory, devising an intervention, and establishing its feasibility and potential efficacy and a real-world clinical environment.
- The pilot is on a small scale, which has implications about the representativeness of our participants. A randomised clinical trial is now required to inform wider implementation.

## Introduction

People previously treated for cutaneous melanoma are at risk of recurrences and developing new primary melanomas.^1^ ^2^ The early detection of these events is one of the key aims of structured follow-up programmes for cutaneous melanoma, and these are supported by guidelines in most countries.^1^ ^3^ ^4^ Delivering effective structured melanoma follow-up to a growing population of eligible people is burdensome to health services.^5^ Furthermore, many recurrences and new primaries occur in the intervals between structured melanoma follow-up visits.^1^ ^6^ In recognition of this, guidelines advocate that patients treated for cutaneous melanoma should be instructed to perform total skin self-examinations (TSSEs), and to conduct these examinations regularly in the intervals between structured follow-up visits.^1^ ^4^

There are reasons to believe that such regular TSSEs performed by people previously treated for cutaneous melanoma could yield marked survival benefits.^7^ ^8^ For example, those who detect their own recurrences may have as much as a 63% reduction in mortality.^9^ Furthermore, a review of the efficacy of skin self-examination for early detection of melanoma found evidence of high specificity (83–97%) for the detection of new lesions.^10^ Sensitivity was lower, but the included studies were not conducted with those previously treated for melanoma. It seems likely, although it cannot be stated with certainty, that a previous diagnosis of melanoma would increase knowledge and awareness with a corresponding increase in sensitivity. There is also some evidence, from a US case control trial and Australian modelling paper, that skin self-examination can reduce the development of advanced disease and facilitate early detection of recurrence by people affected by melanoma.^9^ ^11^ It is hoped that support to perform TSSEs could enable both recurrences and new primaries to be detected at a much earlier stage when a cure may still be possible. The risk of recurrence in cutaneous melanoma is influenced by the stage of disease at diagnosis.^11^ Less intense follow-up regimens have been advocated for those with early-stage disease at diagnosis (stage IA, IB, IIA), and effective and sustained TSSEs could be particularly important in underpinning these.^11^ Equally, however, since all patients treated for cutaneous melanoma are at risk of recurrence, effective TSSEs could be viewed as having a role as an adjunct in follow-up irrespective of the clinical stage at diagnosis.

Despite this, TSSEs education and practice appears suboptimal with 70% of American patients with melanoma indicating that they have never been advised to do it.^12^ We have found similar evidence of under-preparedness to conduct TSSEs in a UK population.^13^

Evidence from randomised trials suggests that people can be appropriately trained to conduct TSSEs.^14–18^ However, it is less clear whether TSSEs, once learned, can be sustained. Recent qualitative evidence suggests that the intention to conduct TSSEs wanes with time.^13^ Digital technologies are becoming more prevalent in society, with a recent report that 49% of UK homes own at least one smartphone, tablet and computer.^19^ More and more people are using personal electronic devices, such as tablets and smartphones, to obtain health information and to interact with healthcare providers.^20^ This paper reports the development, pilot testing and preliminary evaluation of the Achieving Self-directed Integrated Cancer Aftercare (ASICA) intervention, a tablet computer-based application designed to prompt and support TSSE at home by people treated for cutaneous melanoma.

## Developing and piloting the ASICA intervention

### Overview

Our approach was based on the key development activities outlined in the Medical Research Council (MRC) Framework for the development and evaluation of complex healthcare interventions.^21^ ^22^ Our developmental approach comprised a number of activities.

*Generated evidence* on how technology has been used in cancer follow-up, how people with melanoma perceived this technology that could be used to support them to conduct TSSEs, and how to target technology at those patients with the most potential to benefit.

*Identified and developed theory* grounded in Information–Motivation–Behaviour skills (IMB) as an explanatory model combined with Control Theory and Implementation Intentions to underpin the theoretical development of the intervention.^23–27^ The IMB model proposes three requisites for engaging in preventive behaviours: individuals must have access to relevant information; be motivated to act; and be capable and confident (self-efficacious) enough to carry out the behaviour in question. IMB has been used successfully to explain and change health relevant, preventive behaviours; for example, an IMB-based intervention was more effective than information alone in increasing HIV prevention behaviour (condom use) in truck drivers.^23^ ^28^ Control theory, first proposed in 1982, proposes that behaviour is maintained through monitoring and evaluation of the discrepancy between goals and current behaviour via a discrepancy-reducing feedback loop.^25^ ^29^ ^30^ A specific goal (eg, performing TSSE) is compared with current behaviour and if a discrepancy is detected, action is taken to bring behaviour closer in line with the goal. If the behaviour gets closer to the goal in response to feedback, the behaviour persists; however, if the discrepancy is perceived to be too great, the individual may disengage from the behaviour. Interventions based on Control Theory are consistently shown to be effective in changing health-related behaviours in clinical and non-clinical populations.^31^ For example, in a metaregression examining interventions to change health-related behaviours in 122 studies, the most effective interventions included techniques based on Control Theory (self-monitoring goal setting, specifying action goals, feedback and review of goals).^32^ A third model used in the current study concerns ‘implementation intentions’ or ‘action plans’.^26^ ^27^ Action Plans are short ‘if-then’ plans that have been shown to be effective in enabling individuals to achieve their behavioural goals in a wide range of contexts. Thus, IMB theory proposes the factors needed to engage in target behaviour—information, motivation and skills/confidence, and Control Theory and Action Plans indicate the processes necessary to keep the behaviour going (goal prioritisation, feedback, behavioural discrepancy detection), and the techniques that can be used to help individuals achieve and maintain target behaviours. Using these models, the components for a potential intervention were theorised in consultation with experts in behavioural science, and the mechanism for the whole intervention to prompt, record and respond to TSSEs by patients in their own homes was conceptualised and implemented using Behaviour Change Techniques (BCTs).

*Modelled the process* of delivery of the combined components of the intervention. A major challenge to this project was to combine the theory and evidence-based components into a viable intervention, and we used innovative methods to simulate the full intervention. This was carried out using an Experience Laboratory event facilitated by experts where healthy volunteers simulated the processes of the theorised ASICA intervention.

**Figure 1:**
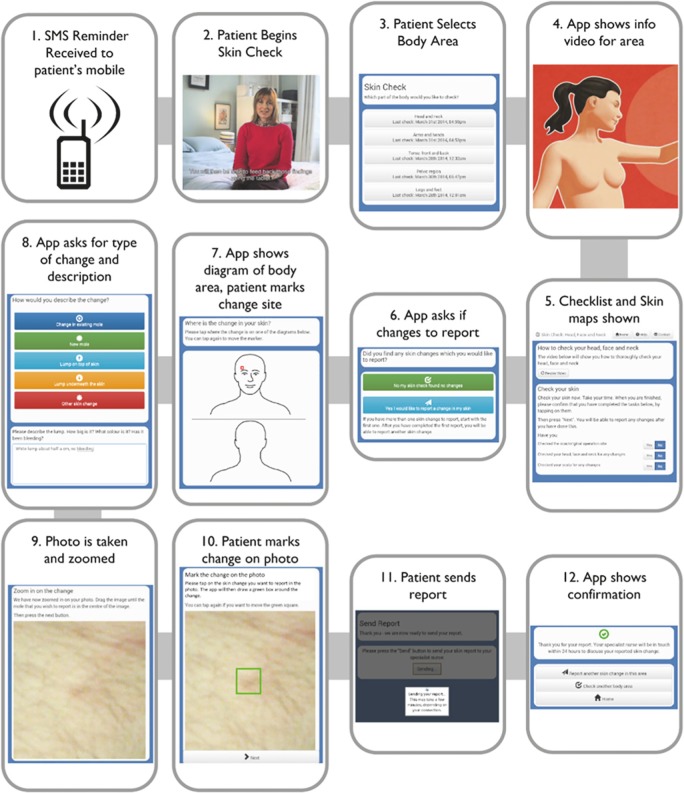
TSSEs procedure as supported by the ASICA application (ASICA, Achieving Self-directed Integrated Cancer Aftercare; SMS, short message service; TSSEs, total skin self-examinations).

Once the prototype ASICA intervention had been developed, we assessed the feasibility and acceptability of the prototype ASICA intervention (figure 1) through a pilot exercise with a group of patients supported by a nurse specialist in dermatology.

## Developing the ASICA intervention

### Generating the evidence to use and target technology

Evidence was derived from three sources. First, a systematic review was conducted to determine how technology has been used to support people with cancer. The methodology and results of this systematic review are reported in detail elsewhere.^33^ Second, interviews were conducted with 21 people previously treated for cutaneous melanoma. Full ethical approval for the interviews was sought from the North of Scotland Research Ethics Committee and granted on 2 May 2012. The methodology and results of these interviews are reported in detail in a previous publication.^13^ Third, clinical data were sought and obtained, where available, on recent recurrences and new primary melanomas diagnosed in Northeast of Scotland. The methods to obtain, analyse and interpret these data have been reported in detail.^6^

When integrated, this evidence suggests that the technology to deliver cancer follow-up care remotely is available, safe and acceptable. Furthermore, people treated for cutaneous melanoma can see the benefit of conducting TSSEs, but feel ill-equipped to perform it properly, safely, regularly and sustainably. They can, however, see the potential of technology to support them in this endeavour and want to be shown how to conduct sequential TSSEs, and then be reminded about when and how to do it. They also believe that this process could be supported by repeated reference to an instructional resource (eg, a video) and self-reference (eg, a digital skin map). Once they have conducted a TSSE, they want to be able to report their findings quickly to a specialist, and be reassured that the specialist would check their report and respond quickly if there were concerns. They would also welcome the potential opportunity to engage with healthcare professionals from their own homes without inconvenience (travel, time off work, parking). This was especially so for rural dwellers.

The evidence garnered from the literature and interviews also found that potential recipients strongly felt that approaches to monitor potential recurrence need to be developed carefully, and should not replace current hospital-based follow-up until their safety and efficacy have been proven. The clinical data also suggested that recurrence is relatively common, occurs early and is usually found at the follow-up clinic within the first year. Therefore, an intervention to support TSSEs should be implemented within a month or so of diagnosis to afford maximum benefit.

### Identifying and developing theory

The research team included an academic general practitioner (GP), a health services researcher, two health psychologists, and two computer scientists. Together, they had expertise in intervention development and evaluation, behaviour change and translating behavioural interventions into programmed computer applications. The chief investigator (an academic GP) first conceptualised the aims, processes and outcomes that the digital intervention should achieve. The final theoretical intervention was then produced in a series of three consensus meetings involving the whole research team.

The overriding aim of the intervention was to prompt the performance and reporting of good quality TSSEs by people previously treated for cutaneous melanoma. To achieve this, individuals must be shown how to use technology to conduct optimal TSSEs and then be prompted to conduct TSSEs regularly. They need to be able to remind themselves how to undertake TSSEs when they are due to do it. The intervention must then transmit the result of each patient's TSSEs to an overseeing clinician, who will then respond appropriately (ie, employ clinical triage) when a patient did identify a concern.

These aims, processes and outcomes were agreed at the first consensus meeting of the whole research team. Consideration was then given to the most appropriate theoretical model able to inform an intervention to achieve these aims, support the necessary processes, and deliver the desired outcomes.

**Figure 2:**
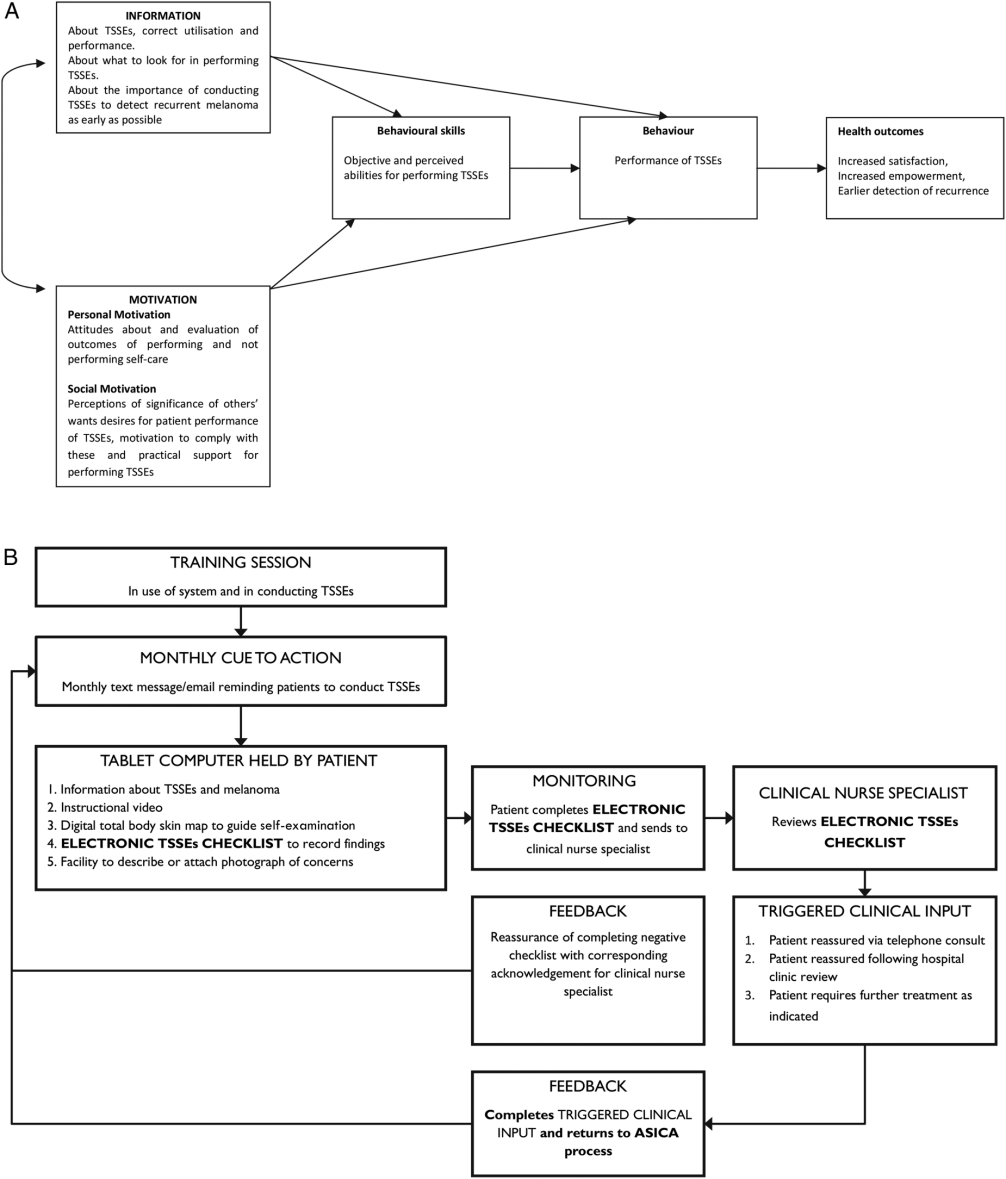
(A) Model demonstrating theoretical processes of ASICA application according to IMB model—adapted from Cowling *et al*.^24^ (B) Schematic demonstrating operationalisation of components and processes of ASICA intervention adapted from Cowling *et al*^24^ (ASICA, Achieving Self-directed Integrated Cancer Aftercare; IMB, Information–Motivation–Behaviour skills; TSSEs, total skin self-examinations).

By consensus with the research team, it was decided that the IMB model offered the most promise in explaining current use of TSSEs.^23^ Using this model, the components for a potential intervention were theorised (ie, components that would provide information about TSSEs, motivate individuals to perform TSSE, and develop skills and confidence to perform TSSE) and the mechanism to prompt, record and respond to TSSEs by patients in their own homes was conceptualised. This is illustrated in figure 2A, B.^23^ ^24^

At a second consensus meeting, the results of the interviews conducted at stage A were considered. It was noted that while the explanatory outline was based on the IMB, the results of interviews A indicated that although patients required more information, they were already highly motivated and we, therefore, required a theory that guided the translation of motivation into action. The psychologists proposed that the process of the intervention should, therefore, incorporate Action Planning and should be revised to be additionally guided by ‘Control Theory’—as this theory deals with the process of self-regulation to change behaviour from a pattern that fails to achieve the person's goal to one that achieves their goal.^23–25^ Together, these theories outline the process of change and give some guidance on the BCTs (ie, the active ingredients that make up an intervention and are required to change behaviour) which the intervention required.^34^ Some techniques were required to develop the knowledge and behavioural skills to enact the behaviour (eg, demonstrating the behaviour, rehearsing/practising TSSEs); some to enhance/maintain the person's motivation to engage in the process of TSSEs (eg, providing information on health consequences of the behaviour (TSSEs), using a credible source for the information); some to enhance confidence that they could conduct TSSEs successfully (eg, mastering the skills necessary); and some to enable self-regulation of action, especially remembering when to act (eg, prompts and cues), and the sequence of actions necessary for the optimal clinical outcome (eg, Action Planning, where patients who have decided to do TSSEs would make a clear plan when, where, and how they would do the examination). Planning ‘how’ to perform TSSE might include involving someone else (eg, to examine areas of skin that they cannot easily see themselves), and planning ‘when’ to receive a reminder. In addition, some techniques were designed to maintain continued engagement in the behaviour (eg, receiving feedback).^26^ ^27^

To produce the final theoretical intervention, a final consensus meeting was held. The whole research team first discussed the fidelity of the theory to the delivery of the intervention, and then worked together to map a theoretical structure for the intervention, incorporating the identified BCTs where appropriate. The intervention demonstrated the target behaviour (with a video clip); enhanced motivation to perform TSSE (with recorded information about the consequences of performing/not performing TSSE); enhanced confidence (with the incorporation of step-by-step instructions and opportunities to try each step in the video clip). The intervention tackled the issue of intentional and non-intentional non-adherence (including forgetting, deferring, avoidance or deciding it is unnecessary) (using cues to prompt individuals to undertake TSSEs); provided individuals with feedback about the behaviour (by sending TSSE results to health professionals and getting the professional reply); and checked adherence to TSSE (by asking individuals to mark skin maps/record how long the personal skin check took). This gave an indication of thoroughness and provides information on those who do it more quickly because, for example, they have other commitments, or those who choose to adopt avoidance. This allows the monitoring of adherence and engagement. A strategy to identify avoidance is very important since without it the clinicians could be making clinical decisions and providing clinical advice based on incorrect information.

### Modelling the process of delivery of the intervention

#### Experience Laboratory event

An Experience Laboratory event was held in May 2013 at Glasgow School of Art's Centre for Design Innovation, in Forres, Moray.^35^ This facility enables the creation of different environments to simulate real-life situations. The processes of delivery for the ASICA intervention, including simulation of the clinical sequences, were developed for use at the event. This included a simulation of the information and TSSEs demonstration for a potential supporting digital application, which was produced and embedded on a hand-held tablet computer, with guidance from experts in design and presentation. Three locations were constructed: a patient's bedroom (see online supplementary photo), a GP’s clinic, and a clinical nurse specialist's office, the latter two being equipped with videoconferencing capability. The intervention components included in the simulation were: the cue to action (ie, the prompt to complete TSSEs); the instructional video (showing how to conduct TSSEs); the skin-map (to be used while conducting TSSEs); and the report sent to health professionals (following completion of TSSEs).

The Experience Laboratory event was facilitated by design experts and attended by five patient volunteers (1 supported by a helper) unaffected by cutaneous melanoma who performed a simulation of the theoretical intervention (as shown in figure 2B), a GP, a clinical nurse specialist in dermatology, and the researchers.

Following an initial briefing session, an existing instructional video produced by MASScot (Melanoma Action and Support Scotland) describing how to conduct TSSEs was viewed by all participants. Two scenarios were constructed and enacted by each of the patient volunteers. In the first scenario, the volunteers were asked to perform TSSEs for which no problems were detected. In the second scenario, the volunteers conducted TSSEs at which a new mole was detected. In this latter scenario, the patients attended the GP surgery location for a video consultation between themselves and the co-located GP, and the remote clinical nurse specialist.

A professional TV company filmed and edited a video of the proceedings. At the conclusion of the day, all participants viewed the video, and a feedback and a debriefing session was held.

#### Integrating components and processes of the ASICA intervention

The Experience Laboratory enabled participating stakeholders to articulate and agree on the benefits which the ASICA intervention could deliver to recipients. Furthermore, the activity enabled the theoretical components of the intervention to be operationalised in the simulation in order to gain insight into how well these integrated and served the purposes for which they were intended, that is, to support the mechanism of prompting, recording, and enabling a response to TSSEs. The Experience Laboratory also enabled the researchers to gain insight into the detailed processes and the sequence in which these should occur to support the effective operation of the ASICA intervention. These were: the language used; training of the user; reporting to the specialist, and receiving feedback from the specialist. The detailed learning achieved on each component is also summarised in online supplementary appendix 1.

#### Combining processes and components in a prototype intervention

As a result of the Experience Laboratory event, the detailed components and processes identified and developed during the theoretical stage were integrated into a prototype ASICA intervention, including a supporting digital application which was designed to run on a Google Nexus 7 tablet computer. Distinct from the application were several other components including:

1. The structured training session required at inception;
2. The initial and recurring cue to action required to remind the patient to conduct a personal skin check; the need for this to be a separate trigger (sent by email or text message to the recipient's mobile phone) was necessary to avoid the risk that the tablet was used only for skin checks with the risk that the prompt would not be received;
3. The specialist's response, a telephone call from the overseeing specialist's nurse within 24 h, since both the human contact and immediacy were perceived as important reassuring factors at a time when a patient could be anxious.

On the basis of the Experience Laboratory findings, the prototype intervention was adjusted for piloting. The need for clear and simple language unifying the application and supporting processes was perceived to be key to user engagement and intervention adherence. Within the digital application, language was made consistent with the language introduced at the training session. This was carried over into an animated instructional video which was produced and divided into chapters based on body areas, and used as a means to demonstrate and remind users about the specific behaviours required to check their body. Conducting the personal skin check using the application was designed to follow a logical sequence supported by a checklist for self-monitoring of completion. The process of feeling for lumps in regional nodal areas was routed so that only the appropriate nodal area was examined by each patient. Patients are also able to check an integrated individualised skin map (formed of a series of professionally produced clinical photographs of each patient) to determine whether skin lesions were new or changing. This function was further supported by the application storing previous reports/images for future reference. At the conclusion of the skin-check, the ASICA application delivers a message that either no problem has been reported or in the event that a symptom concern has been raised, that a specialist will be in touch within 48 h with further advice. In either eventuality, the completion of the TSSEs is recorded and acknowledged giving a sense of completing the processes in a way that provides feedback and reassurance; this acts as a reward for completing the behaviour with the aim of reinforcing the behaviour so that individual patients will keep using the ASICA application.

## Pilot study of the feasibility and acceptability of the prototype ASICA intervention

The prototype ASICA intervention, including the supporting digital application, was subject to a pilot study of feasibility and acceptability among 20 people who had previously been treated for cutaneous melanoma.

### Recruitment

Six practices were purposively selected to represent geographical spread within the National Health Service (NHS) Grampian region of Scotland, and a GP from each was invited to a training meeting to have the protocol explained. The lead GP at each practice identified and approached potential participants for pilot study. Eligible patients were aged over 18 years, had been diagnosed and treated for cutaneous melanoma within the preceding 5 years, were currently receiving hospital-based follow-up, and had no nodal involvement or metastases (ie, in situ to stage 2C). The 20 people agreeing to participate were identified to, and approached by, the research team. The characteristics of participants are shown in table 1. Recruits attended the Medical Illustration Department at the University of Aberdeen to have a full personal body mapping digital photography taken. These were subsequently hosted on a secure server and could be accessed by individual patients to refer to during subsequent skin checks.

**Table 1:** Characteristics of pilot study participants

ID	Age	Gender	Place of residence*	Date of Mel Dx	Site	Stage
001	46	F	Accessible rural	2010	Arm	1.1 mm stage 1B
002	49	F	Other urban area	2012	Knee	0.5 mm stage 1A
003	72	F	Accessible rural	2013	Arm	0.4 mm stage 1A
004	69	M	Urban	2013	Breast	0.8 mm stage 1A
005	62	M	Remote rural	2012	Eyelid	M in situ stage 0
006	66	F	Remote rural	2011	Cheek	0.3 mm stage 1A
007	72	M	Remote small town	2009	Cheek	2.8 mm stage 2A
008	70	M	Remote small town	2012	Shoulder	0.3 mm stage 1A
009*	41	F	Remote rural	2011	Back	>1 mm
010	67	F	Accessible rural	2009	Arm	3 mm stage 2A
011	78	M	Remote small town	2008	Eyebrow	2.6 mm stage 2A
012	42	F	Accessible small town	2011	Back	M in situ stage 0
013	75	F	Accessible rural	2009	Thigh	1.1 mm stage 2B
014	67	M	Accessible rural	2013	Shoulder	2 mm stage 2A
015	46	F	Accessible rural	2011	Abdomen	0.6 mm stage 1A
016	72	M	Accessible rural	2011	Forearm	1 mm stage 1B
017	65	M	Accessible rural	2014	Shoulder	M in situ stage 0
018	69	M	Remote rural	2009	Shoulder	1.5 mm stage 1B
019	44	M	Accessible rural	2012	Abdomen	1.5 mm stage 1B
020	44	F	Accessible small town	2010	Lower leg	0.42 mm stage1A

Classifications from Scottish Government Urban-Rural Classification.^38^

*Staging data were not available for this patient. M, melanoma; Mel Dx, melanoma diagnosis.

### Participant training

Three training sessions (each of 2 h duration) were held in Aberdeen. The meetings followed a structured programme. Participants were introduced to the study and its purposes. The fact that the intervention was experimental (and additional) to their ongoing follow-up was stressed to ensure default from follow-up was not suggested. Participants were instructed in the use of the application and tablet, including how to access their digital skin maps, and their understanding and ability to comply checked. Patients were given detailed instruction manuals for the tablet and the application. The project researcher arranged an individual meeting with one individual who was not able to attend the training sessions. To prepare for a future clinical trial a questionnaire was modified, with permission, from one used previously.^15–17^ The questionnaire (included as online supplementary appendix 2) sought information about respondents’ skin cancer history, their skin self-examination practices and intentions, their attitudes, beliefs, self-efficacy, and intentions about conducting skin self-examination, their Hospital Anxiety and Depression scale, information about comorbidities and their demographic characteristics. Participants were asked to complete the questionnaire on arrival at their initial training session. They were then sent the questionnaire again at the conclusion of the pilot.

### Pilot study process

Participants were sent a monthly email reminding them that it was time to conduct their personal skin check. On receipt of the reminder, it was intended that they would use the ASICA application to help them systematically examine their skin; through the application they were able to view the integrated instructional video chapters which enabled them to do their skin check. A structured electronic report pro-forma was available for completion. Where a new lesion was identified either at the previous melanoma site or at a new one, participants were able to complete a free-text description and/or attach a photograph taken using the tablet's camera function. Completed reports were then sent electronically to a secure and remote server. The returned reports were communicated to and reviewed by an overseeing nurse specialist. Figure 1 illustrates the TSSEs procedure supported by the ASICA application. Patients who had identified concerns were contacted by telephone within 24 h by the reviewing nurse specialist, who either provided reassurance or invited them to an upcoming clinic for subsequent review. At the conclusion of the pilot study, all continuing participants were invited to attend a total skin examination at their GPs’ clinic; 15 accepted this invitation and attended the skin examination at their GP's clinic. Three declined, one because he was on holiday at the time of the appointment, one because he was undergoing treatment for metastatic melanoma. A further one participant did not respond to the invitation.

At the conclusion of the pilot, the project researcher SH contacted all participating patients and the overseeing clinical nurse specialist to conduct a brief telephone interview. These interviews aimed to capture the practical experiences and personal reflection of participants in the pilot study. These were conducted to identify participants’ perceptions of strengths and weaknesses with the components, or the process and delivery of the intervention, so that subsequent improvements could be made. The interviews were guided by a topic schedule. Questions focused on patients’ perceptions of the strengths and weakness of the ASICA application, and how it had functioned. The interviewer also gathered information about how well the technical aspects of the intervention had worked from the nurse-specialist's and patient's perspectives. The interviews were conducted by telephone, and were recorded and transcribed for subsequent analysis and reflection by the research team.

As this was a pilot study, no *a priori* hypotheses were determined based on clinical or psychological outcomes. We did, however, ask participants to complete a questionnaire seeking information about clinical, behavioural, and psychological outcomes to aid preparation for a subsequent clinical trial.

### Pilot study results

#### Feasibility

Details of the number and regularity of the skin checks participants performed during the pilot can be seen in table 2. Of the 20 participants, 15 complied well of whom 8 reported no symptoms during the 6-month pilot and 7 reported at least one issue to the overseeing clinical nurse specialist. Most issues were resolved by submitting further images under the direction of the specialist nurse, with a corresponding telephone call. Two participants subsequently had the lesions spotted during personal skin checks removed, one was a recurrent melanoma and the other was a benign lesion. Of the three less-compliant participants, one regularly checked only his face where his original primary melanoma had been, while another checked selected areas less regularly, citing work pressures and lack of time to conduct TSSEs. Another, a busy mum who stated she found it difficult to make time to conduct a TSSEs, checked her skin only once and on that occasion reported three issues of concern to the overseeing nurse specialist. One participant withdrew from the pilot for undisclosed personal reasons.

**Table 2:** Compliance with intervention and outcome of monthly skin checks

Patient	Month 1 (May)		Month 2 (June)		Month 3 (July)		Month 4 (August)		Month 5 (September)		Month 6 (October)	
	Number of body areas checked	Changes reported	Number of body areas checked	Changes reported	Number of body areas checked	Changes reported	Number of body areas checked	Changes reported	Number of body areas checked	Changes reported	Number of body areas checked	Changes reported
N=8: complied well, reported no symptoms												
P02	5	0	5	0	5	0	5	0	5	0	5	0
P03	0	0	0	0	5	0	5	0	5	0	0	0
P04	5	0	5	0	5	0	0	0	0	0	5	0
P05	5	0	5	0	5	0	5	0	5	0	5	0
P06	5	0	5	0	5	0	5	0	5	0	5	0
P10	5	0	5	0	5	0	5	0	5	0	5	0
P16	5	0	5	0	5	0	5	0	5	0	5	0
P19	5	0	0	0	5	0	0	0	5	0	5	0
N=7: Complied well, reported symptoms												
P01	4	1	0	0	5	2	5	2	4	1	5	0
P07	5	3	5	5	5	2	5	0	5	2	5	0
P08*	4	0	5	0	5	0	5	2	5	0	5	1
P13	3	3	1	1	5	0	5	0	5	0	5	0
P14	0	0	3	2	4	0	5	1	5	0	4	0
P15	5	1	0	0	5	1	5	0	5	0	5	0
P18†	5	1	0	0	5	0	0	0	5	1	0	0
N=3: Complied less well, reported symptoms												
P11‡	1	1	1	1	1	0	1	1	1	1	1	1
P12	0	0	1	0	0	0	3	1	0	0	0	0
P17	0	0	0	0	3	3	0	0	0	0	0	0
N=1: Complied poorly, reported no issues (P20)												
P20	0	0	0	0	0	0	0	0	5	0	0	0
P09 patient withdrew citing personal circumstances making skin checks difficult—not clear what these were												

*P8 diagnosed with recurrent melanoma after excision of lesion noticed during personal skin check.

†P18 diagnosed with benign lesions on both legs after excision of lesions noticed during personal skin check.

‡P11 checked head and neck only.

With respect to the technical operation of ASICA application, the nurse specialist stated that on the few occasions when photographs of new skin lesions had been submitted by participants these were typically of insufficient quality on which to base clinical judgements. However, in almost all cases he was able to contact the patient and direct them to take improved images. As a result, guidelines to take good-quality images have been incorporated into the revised app.

#### Acceptability

Patients were largely positive about their experience of using ASICA application. The user-friendliness of ASICA application was highlighted along with views that participation supported good habits, allowed participants to become familiar with their own bodies, and provided them with empowerment and reassurance. Box 1 describes comments which reflect these themes. Technical issues raised by patients fell into three categories. There were minor issues with the interface (eg, parts of electronic buttons being obscured) which have been modified. Some patients, especially those in the more remote rural areas, were troubled by issues related to their internet connections. These are less easy to resolve, but are likely to be more common in this particular geographical location than in the majority of the rest of the UK. Government initiatives and technological advances will help going forward in this regard. Similarly, there were some issues with the hardware, for example, a malfunctioning charger in one case and a damaged screen in another.

Box 1 **Comments from patient interviews reflecting views on usability and acceptability**

A user-friendly device

P03—“Yes, it was quite clear the actual information that we were given, very clear, beautifully set out, very easy to use and understand.”

P04—“Very good. Very good indeed. It's very clear, easy to understand and useful in tips about parting your hair and getting somebody else to check the back of it for you and things like that, yeah, very clear and easy to understand and you know, tips about how to do awkward places on yourself, yes.”

P05—“So what I've done is have a good look at myself over the preceding days, if you know what I mean, just as and when it was comfortable. And really handy, when I was getting changes, getting up or going to bed or what have you, in the shower. And then just rattle through the app.”

P08—“The animations that were provided I thought were a really good guide, for somebody that's not used to technology it was really simple.”

P17—“Well it tells you exactly what you need to know, there's no question about that.”

P21—“The instructions were excellent, they were very well laid out. The videos were very helpful showing you exactly what you needed to do and how to check yourself all over.”

Establishing good habits

P04—“But the fact that it makes people do it once a month or whatever, it focuses the attention because it's something we'd probably be a bit slapdash with normally.”

P13—“The tablet is great. Totally self-explanatory and the videos are very easy to watch and everything so it very easy to do and send off the report. Everything was great.”

P15—“It made you really thorough about the skin check procedure. There was no way you could miss anything out. It was really good.”

P16—“Yes, as I say, it's all clear and it's really good to see every part of your body…to go through it all in separate stages. Yes, it make you do it all in a through way, which is important, since I'm not getting checked at the hospital anymore, so it's really important that I've got to remember to check my whole body in case something appears.”

Getting to know my own body

P01—“I like having the maps to look at because I've got a lot of moles but I have discovered there might be a blind spot on my arms where it's not really getting my arm—if you know what I mean?”

P15—“Without this it becomes very difficult to remember if anything has changed very much since the last time you looked. This was really the first time I've ever looked really closely at my body, and I think to myself “goodness, I didn't realise I have that there before.” And then I go back to the body map and—which is a salutary exercise in itself—and see “oh yes, it was there.” I suppose it's getting to know your body much better.”

P17—“I never used to think about it, but I know what to look for now. If I see something I know what it is, and what to do. Before, I never would have noticed.”

P21—“The more I've done it over the period of months, the more that I've gotten used to where everything is on my body, where all the different moles are.”

P21—“Before starting this project I probably wasn't really checking my skin that much at all, but since I've been doing this, it's been much more regular and I've been paying much more attention to it.”

Feeling reassured and empowered

P09—“I'm very pleased with it, because it's helping me, you feel in control, that you are looking after yourself.”

P12—“If somebody is checking it, that can get back to you really quickly, then off to the GP. Very re-assuring.”

P14—“And because I was doing it so diligently, I felt good about that.”

P14—“It a brilliant idea, especially for people who are a long way away, because you can do a really thorough check, and received professional reassurance without having to travel all the way to Aberdeen.”

#### Piloting trial procedures

Sixteen participants completed and returned the questionnaire at baseline and outcome. The data are not presented in detail. There were non-significant increases in the proportion of respondents indicating that they intended to check their skin at least monthly, and in the proportion indicating that they would be confident to perform TSSE. No significant changes were observed between baseline and outcome in anxiety, depression or cancer worry. These data will, however, be informative in determining power for a subsequent randomised trial.

## Discussion

### Principal findings

The authors have developed a feasible clinical intervention process based on a digital tablet-based application to prompt, record, and respond to regular TSSE by people previously treated for cutaneous melanoma. This has proven to be acceptable and safe for patients to use. There is also preliminary evidence that it can help reinforce and sustain TSSEs in a way that has not previously been possible. Further, there is some early evidence that it can bring new skin problems under medical attention sooner than would otherwise have been the case. It must also be noted, however, that the fact that a minority of patients did not comply or complied only partially indicates that ASICA application will not compel all patients to conduct regular TSSEs or might require tailoring for some patients.

### Strengths and limitations

#### Strengths

The approach adopted for developing the ASICA intervention had several inherent strengths. Developing interventions that employ digital technologies to deliver aspects of healthcare in a completely new way is immensely challenging. For this reason, our approach benefited from employing the structured, iterative, and well-rehearsed approach advocated by the MRC framework.^21^ ^22^ The use of the Experience Laboratory allowed simulation of the complete intervention, integrating components based on theory and evidence. The experience of the team in following this approach, and the strong theoretical underpinning of the IMB and Control Theory models allowed the project to be phased and focused.^23^ ^24^ We involved key stakeholders—potential patients, clinicians, technology specialists, behavior-change intervention specialists, health service researchers—at each stage of the process so that their perspectives were identified and incorporated throughout. Furthermore, adopting this multidisciplinary approach enabled an ongoing understanding of the full spectrum of potential challenges and caveats which the intervention was required to overcome, complemented by an ability to exploit the enablers perceived by each group. We were also able to ensure that we optimised the potential of the ASICA digital application by identifying the necessary processes and components, and ensuring that these were developed and embedded within the intervention in the most effective way.

#### Limitations

Some limitations must be acknowledged. The pilot was conducted on a small scale within Northeast Scotland. Clearly, this has implications about the representativeness of our participants. In terms of the whole Scottish population, they were relatively affluent and also willing to learn about technology. It was assumed that all patients were physically capable of using the tablet and the application; one patient who could not use the fingers had to be supplied with a stylus. There were other disabilities that were not provided for, for example, poor eyesight, lack of proficiency in English, and restricted physical movement. A range of adherence was observed during the study and we were unable to understand this in detail. ASICA application, as currently configured, will not suit everyone, but it may be possible to tailor it to an individual need. While the developed intervention may have greater value and relevance among people familiar with technological advances, and in localities where the clinical service is delivered to patients remotely located from the clinical centre, it is likely to have utility among a broad range of patients after melanoma diagnosis and treatment. This view is supported by noting that people with melanoma from stage 0 to 2C were willing to take part.

These limitations must be viewed against the backdrop of societal trends to embrace modern technology, and an increasing appetite among clinicians and policymakers to diagnose and manage skin cancer using digital means. A recent review, for example, identified 40 applications—with divergent quality and developmental rigour—for monitoring and diagnosis of pigmented skin lesions.^36^

### Context with other studies

Where interventions have been specifically developed to improve TSSEs practice and subjected to randomised trial, the results have been disappointing, although the recruited patient groups have been different to this pilot study. Two randomised trials, one in a general US primary care population and another in Australian men over 50 years of age, at increased risk but with no previous melanoma, educated using brochure or video demonstrations only, reported increased TSSEs practice for 3–7 months, with participation returning to baseline after 1 year.^14–17^ A further study conducted among US patients referred to a hospital pigmented lesion clinic, reported significant increases in TSSEs practice at 4 months when patients had received a educational module and a personal skin map.^18^ Previous trials are informative to the current intervention for three reasons. First, all three were conducted for patients at increased risk rather than for patients actually treated for melanoma. It is, therefore, likely that the target group of the ASICA intervention will be more motivated to conduct and sustain TSSEs than previously studied groups. Second, previous intervention development provides evidence that several of the components developed using health psychology-based approaches and incorporated into ASICA application (such as the instructional videos, personal skin maps, cues to action and sample photographs) have the potential to promote and sustain, at least in the short-term, TSSEs in patients who form a lower risk group than the ASICA target population.^14–18^ Third, and perhaps most importantly, the interventions previously trialled have comprised one-off educational activities with the issue of videos, booklets or brochures to patients for subsequent personal use.^14–18^ ASICA application, on the other hand, will use familiar everyday technology to prompt and sustain the behaviour over time in the participant's own homes, which should increase the likelihood of success.^37^

### Lessons learned from this study

Evidence for components of previous interventions that have sustained TSSEs in the medium term have been translated onto a theoretical intervention based on well-evidenced theoretical models using the BCTs Taxonomy v1 to implement the active behaviour change mechanisms.^34^ We found that an Experience Laboratory can provide rapid feedback on a developing theoretical intervention, enabling it to be optimised for field testing. Finally, we have used carefully assembled theory and knowledge to build a working prototype of an actual digital intervention to support TSSEs by people previously treated for cutaneous melanoma. This has functioned well in a real-world pilot. It has succeeded in actually supporting, and responding to TSSEs in a group of patients who have appreciated and enjoyed using it. We have found that it is a feasible and desirable intervention. We are now aware about the minor modifications that are required to proceed to a definitive clinical trial employing the ASICA intervention. Such a trial, conducted at several UK centres to ensure wider applicability, should now follow shortly so that we can consolidate the promising findings reported here with definitive evidence of ASICA application's role in future melanoma follow-up.

## References

[1] Marsden JR, Newton-Bishop JA, Burrows L Revised UK guidelines for the management of cutaneous melanoma. Br J Dermatol 2010;163:238–56. 10.1111/j.1365-2133.2010.09883.x 20608932

[2] Bradford PT, Freedman DM, Goldstein AM Increased risk of secondary primary cancer after a diagnosis of melanoma. Arch Dermatol 2010;146:265–72. 10.1001/archdermatol.2010.2 20231496 PMC3076705

[3] Murchie P, Nicolson MC, Hannaford PC Patient satisfaction with GP-led melanoma follow-up: a randomised controlled trial. Brit J Cancer 2010;102:1447–55. 10.1038/sj.bjc.6605638 20461089 PMC2869159

[4] Marciano NJ, Merline TL, Bessen T To what extent are current guidelines for cutaneous melanoma follow up based on scientific evidence? Int J Clin Pract 2014;68:761–70. 10.1111/ijcp.12393 24548269 PMC4238419

[5] Rychetnik L, Morton RL, McCaffery K Shared care in the follow-up of early-stage melanoma: a qualitative study of Australian melanoma clinicians’ perspectives and models of care. BMC Health Serv Res 2012;12:468 10.1186/1472-6963-12-468 23253951 PMC3537530

[6] Auckland RL, Wassell PR, Hall S Exploring patterns of melanoma recurrence in Northeast Scotland to inform the introduction a digital self-examination intervention. BMC Dermatol 2014;14:4 10.1186/1471-5945-14-4 24612627 PMC3984711

[7] Moore-Dalal K, Zhou Q, Panageas KS Methods of detection of first recurrence in patients with stage I/II primary cutaneous melanoma after sentinel lymph node biopsy. Ann Oncol 2008;15:2206–14. 10.1245/s10434-008-9985-z 18512102

[8] Hull P, Piemontesi N, Lichtenwald J Compliance with self-examination surveillance in patients with melanoma and atypical moles: an anonymous questionnaire study. J Cutan Med Surg 2011;15:97–102. 10.2310/7750.2011.10011 21477557

[9] Berwick M, Begg CB, Fine JA Screening for cutaneous melanoma by skin self-examination. J Natl Cancer Inst 1996;88:17–22. 10.1093/jnci/88.1.17 8847720

[10] Hamidi R, Peng D, Cockburn M Efficacy of skin self-examination for the early detection of melanoma. Int J Dermatol 2010;49:126–34. 10.1111/j.1365-4632.2009.04268.x 20465635

[11] Turner RM, Bell KJ, Morton RL Optimizing the frequency of follow-up visits for patients treated for localized primary cutaneous melanoma. J Clin Oncol 2011;29:4641–6. 10.1200/JCO.2010.34.2956 22067399

[12] Korner A, Coroiu A, Martins C Predictors of skin self-examination before and after a melanoma diagnosis; the role of medical advice and patient's level of education. Int Arch Med 2013;6:8 10.1186/1755-7682-6-8 23446040 PMC3599942

[13] Hall S, Murchie P Can we use technology to encourage self-monitoring by people treated for melanoma? A qualitative exploration of the perceptions of potential recipients. Support Care Cancer 2014;22:1663–71. 10.1007/s00520-014-2133-3 24510193

[14] Janda M, Baade PD, Youl PH The skin awareness study: promoting thorough skin self-examination for skin cancer among men 50 years or older. Contemp Clin Trials 2009;31:119–30. 10.1016/j.cct.2009.11.003 19900577

[15] Janda M, Neale RE, Youl P Impact of video-based intervention to improve the prevalence of skin self-examinations in men 50 years or older: the randomized skin awareness trial. Arch Dermatol 2011;147:799–806. 10.1001/archdermatol.2011.48 21422325

[16] Janda M, Youl P, Neale R Clinical skin examination outcomes after a video-based behavioral intervention: analysis from a randomized clinical trial. JAMA Dermatol 2014;150:372–9. 10.1001/jamadermatol.2013.9313 24553807

[17] Lee K, Weinstock M, Risica P Component of a successful intervention for monthly skin self-examination for early detection of melanoma: the ‘check it out’ trial. J Am Acad Dermatol 2008;58:1006–12. 10.1016/j.jaad.2008.03.008 18406492 PMC2464906

[18] Oliveria S, Dusza S, Phelan D Patient adherence to skin self-examination; effect of nurse intervention with photographs. Am J Prev Med 2004;26:152–5. 10.1016/j.amepre.2003.10.006 14751328

[19] Deloitte LLP. Deloitte 8th Annual Media Consumer Survey 2014. The Digital Divide London, 2014 http://www.deloitte.co.uk/mediaconsumer/ (accessed 22 Dec 2014).

[20] Healthcare UK. Digital health: working in partnership. Healthcare UK, Department of Health and UK Trade & Investment. First published, London, 31 January 2013 https://www.gov.uk/government/publications/digital-health-working-in-partnership

[21] Craig P, Dieppe P, Macintyre S Developing and evaluating complex interventions: the new Medical Research Council guidance. Brit Med J 2008;337:979–83. 10.1136/bmj.a1655 PMC2769032 18824488

[22] Moore G, Suzanne Audrey S, Mary Barker B Process evaluation of complex interventions UK Medical Research Council (MRC) guidance. http://decipher.uk.net/process-evaluation-guidance/ (accessed 9 Dec 2014).

[23] Fisher JD, Fisher WA Changing AIDS-risk behavior. Psychol Bull 1992;111:455–74. 10.1037/0033-2909.111.3.455 1594721

[24] Cowling T, Huckvale K, Ratnapalan M Protocol—Self-care apps for asthma. Version 1.4 01/11/2011. http://www.crd.york.ac.uk/PROSPEROFILES/1708_PROTOCOL_20111002.pdf (accessed 5 Jan 2015).

[25] Carver CS, Scheier MF Attention and self-regulation: a control theory approach to human behaviour. New York, USA: Springer, 1981.

[26] Gollwitzer PM Implementation intentions: strong effects of simple plans. Am Psychol 1999;54:493–503. 10.1037/0003-066X.54.7.493

[27] Gollwitzer PM, Sheeran P Implementation intentions and goal achievement: a meta-analysis of effects and processes. Adv Exp Soc Psychol 2006;38:69–119. 10.1016/S0065-2601(06)38002-1

[28] Cornman DH, Schmiege SJ, Bryan A An information-motivation-behavioral skills (IMB) model-based HIV prevention intervention for truck drivers in India. Soc Sci Med 2007;64:1572–84. 10.1016/j.socscimed.2006.11.011 17257724 PMC4675654

[29] Carver CS, Scheier MF On the self-regulation of behavior. New York: Cambridge University Press, 1998.

[30] Carver CS, Scheier MF Self-regulation of action and affect. Handbook of self-regulation: research, theory, and applications, 2004:13–39.

[31] Dombrowski SU, Sniehotta FF, Avenell A Identifying active ingredients in complex behavioural interventions for obese adults with obesity-related co-morbidities or additional risk factors for co-morbidities: a systematic review. Health Psychol Rev 2012;6:7–32. 10.1080/17437199.2010.513298

[32] Michie S, Abraham C, Whittington C Effective techniques in healthy eating and physical activity interventions: a meta-regression. Health Psychol 2009;28:690–701. 10.1037/a0016136 19916637

[33] Dickinson R, Hall S, Bond CM Using technology to deliver cancer follow-up: a systematic review. BMC Cancer 2014;14:311 10.1186/1471-2407-14-311 24885758 PMC4101828

[34] Michie S, Richardson M, Johnston M The behavior change technique taxonomy (v1) of 93 hierarchically clustered techniques: building an international consensus for the reporting of behavior change interventions. Ann Behav Med 2013;46:81–95. 10.1007/s12160-013-9486-6 23512568

[35] Computescotland.com website. http://www.computescotland.com/distance-lab-forres-joined-by-centre-for-design-innovation-3709.php (accessed 22 Dec 2014).

[36] Kassianos APL, Emery JD, Murchie P Smartphone applications for melanoma detection by community, patient and generalist clinician users: a review. Brit J Dermatol 2015;172:1507–18. 10.1111/bjd.13665 25600815

[37] Consolvo S, McDonald DW, Landay JA *Theory-driven design strategies for technologies that support behavior change in everyday life* CHI 09 Proceedings of the SIGCHI Conference on Human Factors in Computing Systems 2009:405–14.

[38] Scottish Government Urban Rural Classification. The Scottish Government 2012 http://www.scotland.gov.uk/Topics/Statistics/SIMD/SIMDPostcodeLookup (accessed 6 Feb 2012).

